# Photoluminescence and Crystal-Field Analysis of Reddish CaYAl_3_O_7_: Eu^3+^ Phosphors for White LEDs

**DOI:** 10.3390/ma18071578

**Published:** 2025-03-31

**Authors:** Zhaoyu Li, Da Yi, Tianpei Xu, Yong Ao, Weiqing Yang

**Affiliations:** 1 School of Computing and Artificial Intelligence, Southwest Jiaotong University, Chengdu 610031, China; lizhaoyu@swjtu.edu.cn; 2 China Railway Engineering Group Limited, Beijing 100039, China; yida3@crecg.com; 3 Key Laboratory of Advanced Technologies of Materials (Ministry of Education), School of Materials Science and Engineering, Southwest Jiaotong University, Chengdu 610031, China; tianpeixu@my.swjtu.edu.cn (T.X.); yy235yv@163.com (Y.A.)

**Keywords:** white LEDs, fluorescent spectra, complete diagonalization method, CaYAl_3_O_7_: Eu^3+^

## Abstract

Red melilite structure CaY_1−*x*_Al_3_O_7_: Eu*_x_* (*x* = 0.04–0.24) phosphors for white LEDs were synthesized through a straightforward solid-state reaction process. These phosphors exhibit efficient excitation under near-ultraviolet light at 398 nm (^7^*F*_0_ → ^5^*L*_6_), producing the desired emission peak at 622 nm from the transitions of ^5^*D*_0_ → ^7^*F*_2_. The Eu doping concentration was also optimized as *x* = 0.16. The complete 3003 × 3003 energy matrix was constructed based on an effective Hamiltonian including both free-ion and crystal-field interactions within a complete diagonalization method (CDM). Eighteen experimental fluorescent spectra for Eu^3+^ ions at the Y^3+^ site of CaYAl_3_O_7_ crystal were quantitatively identified with high accuracy through fitting calculations. The fitting values are in reasonable agreement with the experimental results, thereby showcasing the efficacy of the CDM in probing luminescent phosphors for white LEDs.

## 1. Introduction

Over the past few years, white light-emitting diodes (w-LEDs) have emerged at the forefront of next-generation lighting technology due to their superior features such as higher brightness, longer operation time (>100,000 h), and lower energy consumption [1,2,3,4,5,6,7,8,9]. Presently, there are two main ways to assemble commercial white LEDs. The first is the combination of GaN-based blue LED chips with yellow- and red-emitting phosphors [10,11,12]. This type of w-LED typically exhibits a low color-rendering index, which is attributed to the relatively strong blue light of blue LED chips and the comparatively low luminescence efficiency of red phosphors. Another method for generating white light involves utilizing GaAIN-based ultraviolet (UV) LEDs coated with three distinct phosphors emitting blue, green, and red wavelengths [13,14,15,16]. But there is still a big challenge in achieving high performance due to the relatively low luminescence efficiency of red-emitting phosphors [17]. As an attempt, various Eu^3+^ doped red phosphors were developed [2,11]. Although these phosphors can effectively overcome the poor color rendering of GaN-based (about 460 nm) white LEDs, they are more difficult to be excited by GaAIN-based near-UV chips at about 400 nm. Moreover, compared with the inherently excessive blue light of a GaN-based chip, near-UV-based w-LEDs should be a more effective packaging model for resolving the poor color rendering of w-LEDs [15,16]. As we know, the Eu^3+^-doped red phosphors can be excited by both blue (460 nm) and near UV (400 nm). Mostly, blue light is more effective than near-UV light because of the crystal-field interaction of host crystals [2]. For this reason, the crystal structure of host crystals can be used to effectively tune the excited spectra of Eu^3+^-doped red phosphors for near-UV light excited w-LEDs.

**Figure 1:**
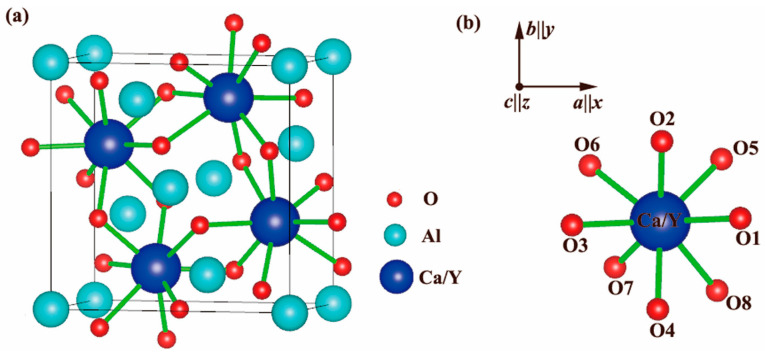
Crystal structure of host crystal CaYAl_3_O_7_: (**a**) ABC_3_O_7_ crystal structure in general form. (**b**) The Ca/Y in the polyhedron with a coordination number of eight.

Recently, the melilite structure CaYAl_3_O_7_ (CYA) [18,19] was demonstrated to be an effective host crystal material for tuning the excitation spectra of red Eu^3+^-doped phosphors for w-LEDs [20,21,22]. The melilite group comprises minerals characterized by a general form ABC_3_O_7_, where both A and B occupy the 4e positions [18,19,20,21,22] (Figure 1a). As shown in Figure 1b, the Ca/Y in the polyhedron with a coordination number of eight will likely be replaced by Eu^3+^ [20,21,22]. Several studies successfully tuned the strongest excitation spectra from blue 460 nm to near-UV 400 nm by partly substituting trivalent Eu^3+^ ions for Y^3+^ ions. The red emission spectra mainly focused on the range of 613–618 nm [20,21,22]. However, the underlying luminescent mechanisms of red Eu^3+^-doping CYA phosphors have been ambiguous to date, and it is desirable to quantitatively identify these spectra.

Here, we demonstrated reddish Ca_1−*x*_YAl_3_O_7_: Eu*_x_* (*x* = 0.04–0.24) (CYAE) phosphors for w-LEDs by using the trivalent Eu^3+^ ion to replace trivalent Y^3+^ ion at the same 4e position of the host crystal. The highest peak of the red emission spectra was red-shifted to 622 nm. In addition, the complete diagonalization (of energy matrix) method (CDM) of crystal-field theory (CFT) was employed to accurately discern the spectra of Eu^3+^ ions within crystals. This involved utilizing an effective Hamiltonian incorporating both free-ion and crystal-field interactions. The as-grown reddish CYAE phosphors exhibit robust red emission spectra, predominantly centered around 622 nm, upon excitation by near-UV light at 398 nm. More importantly, the origins of all experimental 18 emission spectra were accurately identified by the CDM method, which is useful for elucidating the fluorescent mechanism of Eu^3+^ ions at the Y^3+^ site of CYA crystals, particularly in the context of rare earth ion-doped phosphors tailored for applications in w-LEDs.

## 2. Experimental Methods

The reddish CYAE phosphors were synthesized via a straightforward solid-state reaction method. CaCO_3_ (99.5%), Y_2_O_3_ (99.99%), Al_2_O_3_ (99.99%), and Eu_2_O_3_ (99.99%) were selected as the source materials. These starting materials, in appropriate stoichiometric ratios, were thoroughly mixed in an agate mortar to ensure homogeneity. Subsequently, the mixture underwent pre-annealing at 500 °C for 3 h followed by annealing at 1500 °C for 5 h.

The crystal structures of the red CYAE phosphors were analyzed using the XPert Pro MPD (Malvern PANalytical, Almelo, The Netherlands) X-ray diffractometer, employing Cu Kα1 radiation (*λ* = 0.154 nm). The morphology and stoichiometry of the as-grown reddish-orange phosphors were assessed via scanning electron microscopy (SEM, FEI QUANTA FEG 250, Hillsboro, OR, USA) and energy-dispersive X-ray spectroscopy (EDS, FEI QUANTA FEG 250, Hillsboro, OR, USA), respectively. Additionally, their room-temperature photoluminescent (PL) spectra were examined utilizing a Hitachi F7000 spectrofluorometer (Hitachi High-Technologies Corporation, Tokyo, Japan), with a 150 W xenon lamp serving as the excitation energy source.

The calculated energy levels in Table 3 are obtained via the complete diagonalization method (CDM) based on crystal-field theory. An effective Hamiltonian, incorporating free-ion and crystal-field interactions, is constructed as a 3003 × 3003 matrix. The matrix is diagonalized to yield energy levels. Free-ion parameters and crystal-field parameters are optimized by minimizing the root mean square deviation (RMSD) between calculated and experimental spectra. The optimized parameters are then used to compute the energy levels listed in the Calc. column.

## 3. Results and Discussion

### 3.1. Crystal Structure and Morphology Characterization

**Figure 2:**
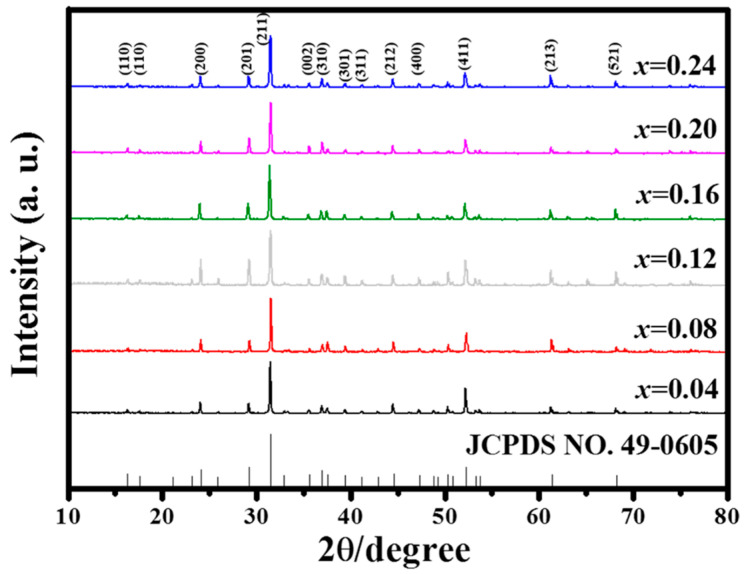
XRD patterns of CaY_1−*x*_Al_3_O_7_: Eu*_x_* phosphors with different Eu concentrations.

**Figure 3:**
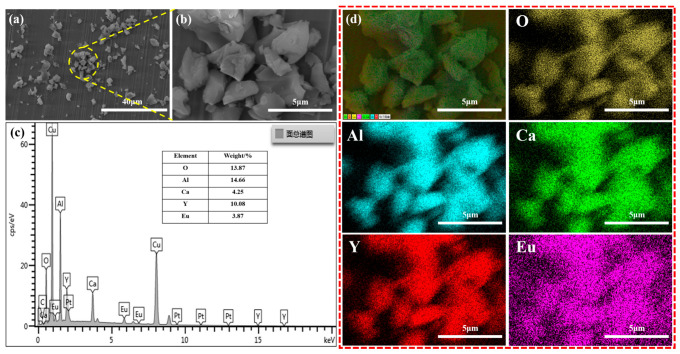
Surface micrograph of CaY_0.76_Al_3_O_7_: Eu_0.24_ phosphors: (**a**) SEM, (**b**) enlarged SEM, and (**c**,**d**) EDS maps (The non-English words in Figure 3c represent: the total spectrum of maps; The non-English words in Figure 3d represent: the electron image).

All XRD patterns of the reddish CYAE phosphors are shown in Figure 2 and are in good agreement with JCPDS No. 49-0605 in the Inorganic Crystal Structure Database. This suggests that Eu^3+^-doped phosphors do not contain any other phases in the host structure. Moreover, the luminous and heat dissipation efficiencies of phosphors intended for white LEDs are frequently influenced by their size and shape, which were, therefore, investigated with SEM. As shown in Figure 3a,b, the CYAE crystalline grains have a diameter of around 2–7 µm, which is appropriate for encapsulation in white LEDs [2,3,11]. Additionally, EDS mapping was utilized to confirm the nominal stoichiometry of the red phosphors CaY_0.76_Al_3_O_7_:Eu_0.24_ (Figure 3c,d). The element mass ratio *M*_Ca_:*M*_Y_:*M*_Al_:*M*_O_:*M*_Eu_ (0.9:0.95:4.59:7.33:0.22) is close to the stoichiometry of CaY_0.76_Al_3_O_7_:Eu_0.24_ (1:0.76:3:7:0.24). The EDS mapping indicates that Eu^3+^-doping ions are uniformly dispersed in the CYAE phosphors.

### 3.2. Photoluminescence Characteristics

**Figure 4:**
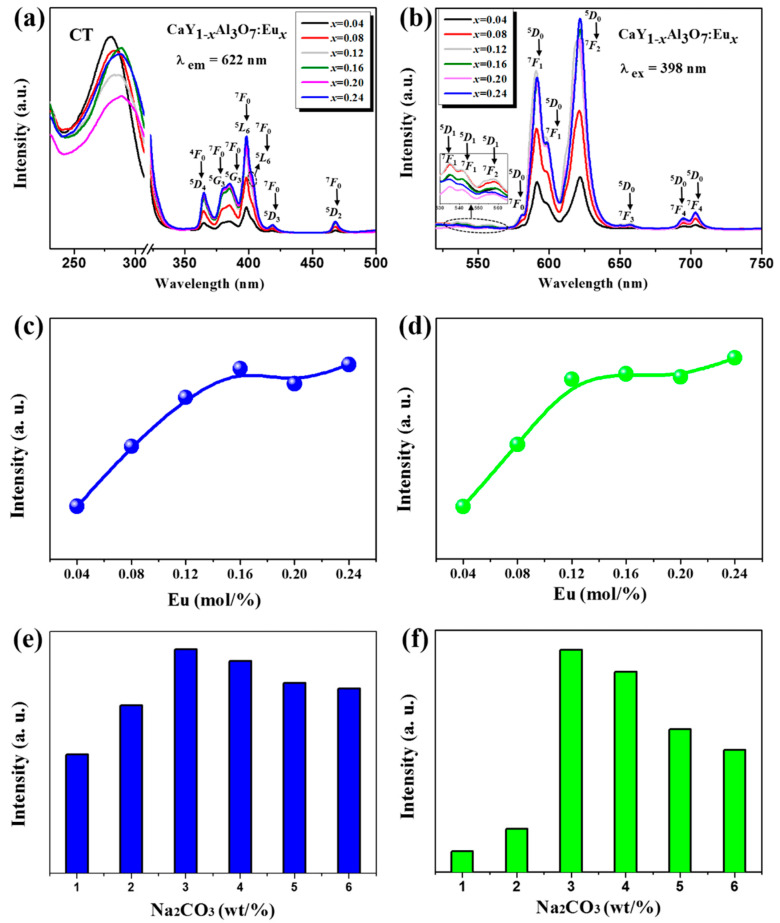
Excitation (**a**) and emission (**b**) spectra of CaY_1−*x*_Al_3_O_7_: Eu*_x_* (*x* = 0.04, 0.08, 0.12, 0.16, 0.20, 0.24) phosphors with emission wavelength 622 nm and excitation wavelength 398 nm; excitation (**c**) and emission (**d**) intensities of CaY_1−*x*_Al_3_O_7_: Eu*_x_* with the various Eu^3+^ doping concentrations, excitation spectra of CaY_1−x_Al_3_O_7_: Eu_x_ phosphors measured at an excitation wavelength of 398 nm, emission spectra of CaY_1−x_Al_3_O_7_: Eu_x_ phosphors measured at an emission wavelength of 622 nm; excitation (**e**) and emission (**f**) intensities of CaY_0.76_Al_3_O_7_: Eu_0.24_ with the various Na_2_CO_3_ doping concentrations.

Figure 4 details the photoluminescent properties of as-grown CYAE phosphors, including the optimal 3 wt% Na_2_CO_3_. In Figure 4a, the excitation spectra of the reddish CYAE phosphors precisely correspond to an emission wavelength of approximately *λ*_em_ = 622 nm. These spectra, characterized by a series of distinct peaks, originate from the *f*-*f* absorption of Eu^3+^ ions situated at the Y^3+^ site within the CaYAl_3_O_7_ crystal lattice. Notably, within these excitation spectra, a characteristic peak centered at the excitation wavelength of *λ*_ex_ = 398 nm was observed, attributed to the ^7^*F*_0_ → ^5^*L*_6_ transition [16]. This observation unequivocally underscores the necessity for the excitation wavelength of the as-grown phosphors to align closely with the near-UV-light LED chips. Moreover, the intensity of this near-UV light is about five times more than that of blue excitation light 460 nm ^7^*F*_0_ → ^5^*L*_6_, obviously showing that the as-grown CYAE phosphors should be more effectively excited by near-UV light. This suggests that the crystal-field interaction of CYA would essentially tune the excited spectra of Eu^3+^ ions at the Y^3+^ site of CaYAl_3_O_7_ crystal. Furthermore, the broadband observed in the PLE spectra ranging from 220 to 300 nm stems from the CT of Eu^3+^-O^2−^ in host crystal CYA [20,21,22]. Additionally, the other existing spectra centered at 365 nm (^7^*F*_0_ → ^5^*D*_4_), 380 nm (^7^*F*_0_ → ^5^*G*_3_), 385 nm (^7^*F*_0_ → ^5^*G*_3_), 403 nm (^7^*F*_0_ → ^5^*L*_6_), 419 nm (^7^*F*_0_ → ^5^*D*_3_), and 468 nm (^7^*F*_0_ → ^5^*D*_2_) were identified by the CDM method. Please see the detailed theoretical analysis in the next sections.

**Figure 5:**
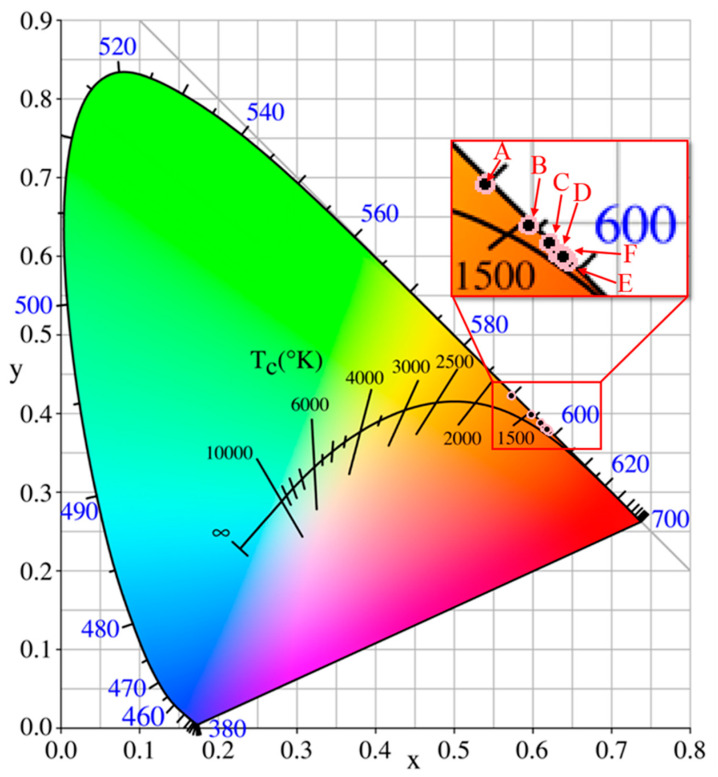
CIE chromaticity map of CaY_1−*x*_Al_3_O_7_: Eu*_x_* phosphors (*x* = 0.04 (A), 0.08 (B), 0.12 (C), 0.16 (D), 0.20 (E), and 0.24 (F)) with near-UV light 398 nm.

As depicted in Figure 4b, upon excitation at 398 nm, the as-grown phosphors exhibit numerous sharp emission peaks originating from Eu^3+^ ions within the host crystals CYA. These emission peaks are centered at wavelengths of 535, 541, 557, 581, 592, 599, 612, 622, 657, 695, and 703 nm. Among them, the main peaks centered at the red right of 622 nm should be ascribed to ^5^*D*_0_ → ^7^*F*_2_ transitions of Eu^3+^ [18]. Therefore, the crystal field of the host CYA crystal has successfully red-shifted toward the desirable red light of 622 nm from the common 613–616 nm of other Eu^3+^-doping host crystals. In addition, the other emission spectra have been identified by CDM (please see Section 3.3). Furthermore, the optimal Eu^3+^-doped concentration of as-grown phosphors CaY_1−x_Al_3_O_7_: Eu_x_ should be proved to be *x* = 0.16, as shown in Figure 4c,d. Additionally, Na_2_CO_3_ was applied as a latent solvent to enhance the photoluminescence properties of as-grown phosphors, as shown in Figure 4e,f. The higher doping concentration (x = 0.24) is favorable for exploring the effect of higher concentrations of Na_2_CO_3_ on the photoluminescence properties. The results show that the optimal doping concentration of Na_2_CO_3_ is 3 wt%. Also, it should be noted that for consistency, the same excitation wavelength (398 nm) and emission wavelength (622 nm) were used. In addition, as illustrated in Figure 5, the CIE chromaticity coordinates progressively shift toward the desired red hue with an increase in the Eu-doped concentration. This observation indicates that the Eu-doped concentration can effectively adjust the CIE chromaticity coordinates of the as-grown phosphors. The observed shifts are both instrumentally measurable and perceptible to the human eye, which highlights the potential for these phosphors to meet specific color requirements in white LED applications. Comparing these results with existing phosphor standards, the ability to adjust the emission color through Eu^3+^ concentration tuning is of practical significance, as it can enhance the performance of these phosphors in lighting applications by improving color rendering and achieving desired color outputs.

### 3.3. Crystal Field Analysis

A commonly employed approach for computing the optical spectroscopy of rare earth ions in crystals relies on parametric modeling calculations [23], which are considered more practical and less labor-intensive compared to prevalent first-principles methods [24,25,26]. In this study, the effective Hamiltonian for the CaYAl_3_O_7_: Eu^3+^ phosphor system can be succinctly expressed as Equation (1):

(1) $$H=H_{FI}+H_{CF}$$

where the free-ion Hamiltonian *H_FI_* can be explicitly expressed as Equation (2):

(2) $$H_{FI}=E_{ave}+\sum_{k=2,4,6}F^{k}{\hat{f}}_{k}+\zeta_{4f}{\hat{A}}_{SO}+\alpha {\hat{L}}^{2}+\beta \hat{G}(G_{2})+\gamma \hat{G}(R_{7})+\sum_{i=2,3,4,6,7,8}T^{i}{\hat{t}}_{i}+\sum_{j=0,2,4}M^{j}{\hat{m}}_{j}+\sum_{k=2,4,6}P^{k}{\hat{p}}_{k}$$

The significance of each interaction operator and its coefficient in Equation (2) has been extensively elaborated upon in various reviews and monographs [27,28,29]. The specific configuration of the crystal-field Hamiltonian H_CF_ in Equation (1) heavily relies on the local site symmetry surrounding the central ion. In the CaYAl_3_O_7_ crystal, the Y^3+^ host ion is potentially substituted by the Eu^3+^-dopant ion, surrounded by the eight nearest oxygen ions.

The site symmetry for this [YO_8_] cluster is categorized under the C_s_ point group. However, the inclusion of both free-ion parameters and independent phenomenological ones for *H_CF_* with Cs symmetry would lead to a significantly larger number than that of the experimental optical spectra in this study, potentially leading to an ‘over-fit’ problem [30] in subsequent calculations.

Therefore, we approximate the C_s_ symmetry with D_4h_ symmetry. This adjustment is supported by the rationale illustrated in Figure 1. Such an approach, known as the ascent/descent in symmetry (ADS) method, has been commonly employed in crystal-field modeling for rare earth ion-doped crystals [31,32,33].

Thus, under tetragonal D_4h_ symmetry, the crystal-field interaction *H_CF_* in Equation (1) *H_CF_* can be written, in the Wybourne notation, as Equation (3) [27,29]:

(3) $$H_{CF}=B_{20}C_{20}+B_{40}C_{40}+B_{44}(C_{44}+C_{4,-4})+B_{60}C_{60}+B_{64}(C_{64}+C_{6,-4})$$

where *B*_kq_ are crystal-field parameters (CFPs) and *C*_kq_ are the Racah spherical tensor operators. Typically, the experimental optical band positions for the CaYAl_3_O_7_: Eu^3+^ phosphor are determined by diagonalizing the complete 3003 × 3003 energy matrix of the Hamiltonian *H* in Equation (1). This diagonalization is performed based on the basic set of commonly used multiplets ^2*S*+1^*L_J_*. Furthermore, each parameter, which characterizes the strength of the interaction in Equations (2) and (3), is determined through fitting procedures aimed at minimizing the root mean square (rms) deviation σ between experimental and calculated optical spectra [23]. In our experiments, only eighteen intraconfigurational f−f transitions are observed. Consequently, we consider dominant free-ion parameters *E*_ave_, Coulomb repulsions (*F*^2^, *F*^4^, *F*^6^), spin–orbit parameter *ζ*_4f,_ and five CFPs *B*_kq_ as adjustable variables, while other secondary parameters are fixed at their mean values as provided in [27]. Therefore, the fitting calculations aim to find the global minimum in the ten-dimensional parameter space.

It is important to note that the initial values of the CFPs significantly influence the final fitting results [21], and these values can be reasonably estimated using the superposition model (SPM). The SPM relies on the approximate spherical polar coordinates of eight oxygen ligands provided in Table 1, along with the intrinsic CFPs of Eu^3+^ ions with eight-fold coordination [2].

All the best-fitted parameters, with a σ value of 26.7 cm^−1^, are listed in Table 2. Additionally, the calculated optical spectra are listed and compared with the experimental spectra in Table 3. It is worth mentioning that only the multiplets to which the experimental optical spectra are assigned are included in the list.

**Figure 6:**
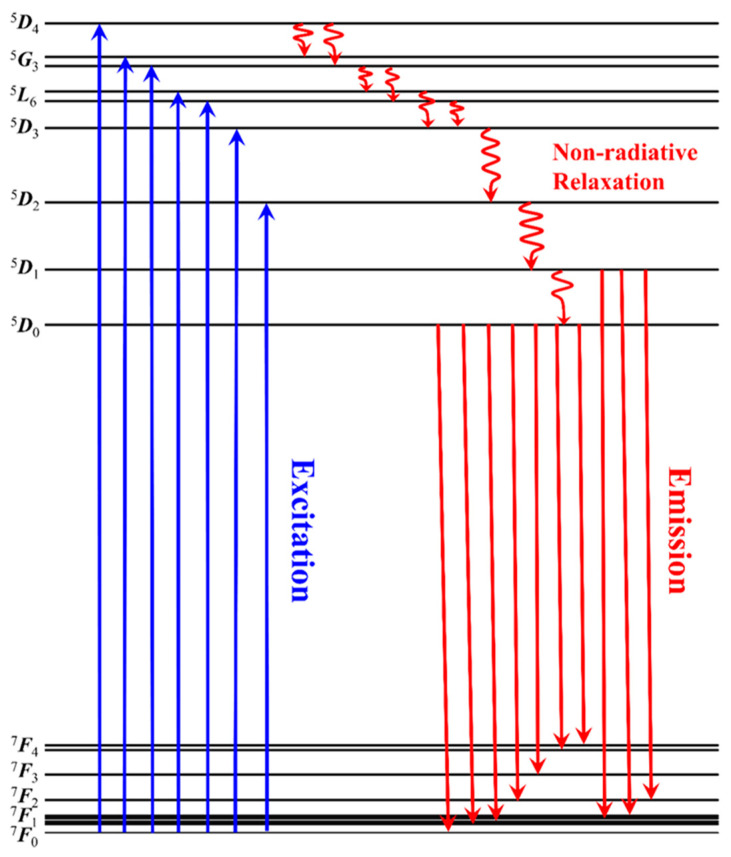
Luminescence mechanism of Eu^3+^ ion-doped CaYAl_3_O_7_ phosphors for white LEDs.

Table 3 and Figure 6 illustrate that the calculated optical spectra of CaYAl_3_O_7_: Eu^3+^ phosphors exhibit reasonable agreement with the experimental spectra, which suggests that the crystal-field modeling effectively explains the excitation and emission spectra of the phosphors under study. However, there are large standard deviations observed in the free-ion parameters listed in Table 2 and discrepancies between the calculated and experimental values presented in Table 3. Two potential reasons for these disparities are identified: (i) First is the limited number of observed optical band positions available for fitting calculations, as all optical spectroscopy experiments were conducted at room temperature. Obtaining additional optical spectra at cryogenic temperatures could enhance the accuracy of the fitting results. (ii) The substitution of the crystal field with D_4h_ symmetry for the actual C_s_ symmetry introduces additional error.

## 4. Conclusions

In summary, reddish CYAE phosphors suitable for white LEDs were synthesized using a straightforward solid-state reaction approach. When excited by near-ultraviolet light (398 nm), these phosphors exhibit highly efficient emission, producing an intense reddish light centered at 622 nm. The complete 3003 × 3003 energy matrix of Eu^3+^ ions located at the approximately tetragonal (*D*_4h_) Y^3+^ site within the melilite structure of CaYAl_3_O_7_ crystal was successfully utilized to quantitatively identify the corresponding spectra through a diagonalization method, marking a pioneering achievement. This novel approach provides insights into the luminescent mechanisms of Eu^3+^-doped phosphors for w-LEDs. It holds promise for similar applications with phosphors doped with other rare earth elements, thereby facilitating the advancement and utilization of innovative fluorescent materials.

## Figures and Tables

**Table 1:** The spherical polar coordinates (*R*, *θ*, *ϕ*) of eight oxygen ligands for [YO_8_] cluster expressed in the coordinate system shown in Figure 1 for both the actual C_s_ and approximate D_4h_ symmetry.

Ligand	[YO_8_]-C_S_			[YO_8_]-D_4H_		
	R (nm)	*θ* (°)	∅ (°)	R (nm)	*θ* (°)	∅ (°)
O1	2.524	67.57	−4.12	2.460	60.44	0
O2	2.397	53.32	90.85	2.460	60.44	90
O3	2.397	53.32	179.15	2.460	60.44	180
O4	2.521	67.54	−85.95	2.460	60.44	−90
O5	2.789	123.28	34.55	2.588	123.79	45
O6	2.394	116.34	135.06	2.588	123.79	135
O7	2.789	123.28	−124.55	2.588	123.79	−135
O8	2.38	132.26	−45	2.588	123.79	−45

**Table 2:** The best-fitted free-ion parameters and CFPs (in cm^−1^) for Eu^3+^ ion in CaYAl_3_O_7_ phosphors for white LEDs.

E_ave_	F^2^	F^4^	F^6^	ζ_4f_	B_20_	B_40_	B_44_	B_60_	B_64_
63,302	80,351	68,113	37,363	1260	−669	2668	1283	2265	2422

**Table 3:** The calculated and experimental energy levels (in cm^−1^) of Eu^3+^ ion in CaYAl_3_O_7_ phosphor.

Multiplet	Cal c.	Expt.	∆E	Multiplet	Cal c.	Expt.	∆E
^7^F_0_	57	0	−57	^5^D_3_	23,894	23,878	−16
^7^F_1_	294	303	9		23,897		
	462	506	44		23,934		
^7^F_2_	824				23,968		
	947				23,985		
	1060	1050	−10	^5^L_6_	24,441		
	1119	1123	4		24,515		
^7^F_3_	1616				24,521		
	1781				24,539		
	1868				24,639		
	1880				24,765	24,789	24
	1969	1990	21		25,009		
^7^F_4_	2375				25,115	25,113	−2
	2415				25,206		
	2855	2809	−46		25,316		
	2871			^5^G_3_	25,599		
	2943	2981	38		25,695		
	3204				25,760		
	3263				25,835		
^5^D_0_	17,208	17,206	−2		25,961	25,961	0
^5^D_1_	18,963	18,994	31		26,302	26,274	−28
	18,985	19,004	19		26,800		
^5^D_2_	21,321				27,002		
	21,333				27,193		
	21,386	21,358	−28		27,235		
	21,388				27,300		
					27,421	27,421	−9

## Data Availability

Data will be made available on reasonable request.
